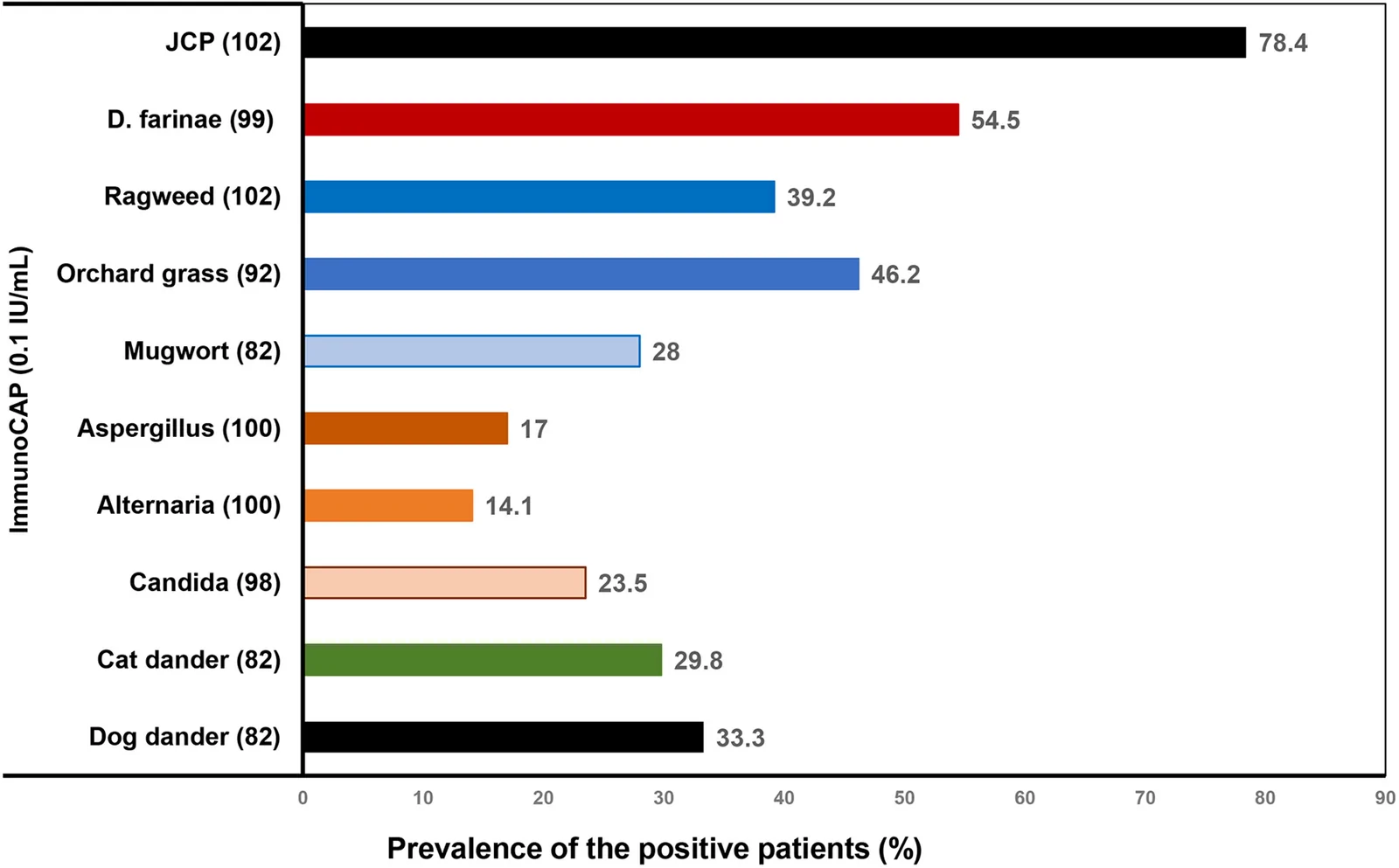# Clinical utility of Oriton IgE CHEMIPHAR™ for predicting skin prick test positivity in patients with allergic airway diseases^☆^

**DOI:** 10.1016/j.waojou.2026.101479

**Published:** 2026-11-02

**Authors:** Erika Naito, Tomoyuki Soma, Kazuyuki Nakagome, Ryu Sekiya, Yoshitaka Uchida, Takehito Kobayashi, Makoto Nagata

**Affiliations:** a Department of Respiratory Medicine, Saitama Medical University, Iruma-gun, Saitama 350-0495, Japan; b Allergy Center, Saitama Medical University, Iruma-gun, Saitama 350-0495, Japan

**Keywords:** Allergic airway disease, CHEMIPHAR, IgE, ImmunoCAP, Skin prick testing

## Abstract

**Introduction:**

Skin prick testing (SPT) and the measurement of serum allergen-specific IgE antibodies are common diagnostic tools for allergic diseases. Oriton IgE CHEMIPHAR™ (CHEMIPHAR) is a simple and convenient serological assay for assessing allergen-specific IgE antibody levels that enables rapid clinical decision-making. However, its clinical application remains limited. The current study evaluated the analytical performance and clinical relevance of CHEMIPHAR in identifying SPT positivity.

**Methods:**

Patients with allergic airway diseases were assessed for SPT positivity and serum allergen-specific IgE antibody levels against 10 common aeroallergens in Japan. Serum-specific IgE antibodies were measured using CHEMIPHAR and ImmunoCAP. The concordance between CHEMIPHAR and SPT was evaluated and compared with that between ImmunoCAP and SPT.

**Results:**

In 168 eligible patients, 168 CHEMIPHAR assays, 139 SPTs, and 108 ImmunoCAP assays were performed. The positivity rate for Japanese cedar pollen (JCP) was the highest across all methods, followed by *Dermatophagoides farinae* (*Df*). Meanwhile, the positivity rate for *Aspergillus* and *Alternaria* was low. The positivity rates for allergens with moderate prevalence varied among the methods. Overall, the concordance with SPT was high for both assay systems for most allergens, except for cat dander. The sensitivity for SPT positivity varied by allergen, with high sensitivity observed for JCP, *Df*, and orchard grass, and lower sensitivity for pet dander in both systems, and for fungal allergens with CHEMIPHAR compared with ImmunoCAP. The specificity for JCP was higher with CHEMIPHAR than with ImmunoCAP. Meanwhile, the specificity for the remaining allergens was similarly high with both systems. CHEMIPHAR showed significant AUC values for a greater number of inhalant allergens than ImmunoCAP. The Kappa coefficients confirmed the agreement with SPT, ranging from substantial agreement for JCP and *Df* with CHEMIPHAR and for *Df* with ImmunoCAP, to fair agreement for three fungal allergens and cat dander with CHEMIPHAR and for *Candida* with ImmunoCAP.

**Conclusion:**

CHEMIPHAR had a clinically relevant performance in identifying allergen sensitization, and it showed concordance with SPT comparable with that of ImmunoCAP. Based on these findings, CHEMIPHAR can be a practical alternative assay for diagnosing allergen sensitization.

## Introduction

Allergic diseases are IgE-mediated disorders caused by sensitization to specific allergens. Exposure to causative allergens can cause or exacerbate various allergic diseases, including anaphylaxis, food allergy, allergic rhinitis (AR), and asthma. Seasonal exposure to aeroallergens increases the risk of asthma and rhinitis exacerbations, characterized by deteriorated pulmonary function, worsening symptoms, and elevated lipid mediator levels in exhaled breath condensates in sensitized individuals.^1^, ^2^, ^3^, ^4^, ^5^ Sensitization to perennial allergens contributes to chronic pathophysiology, such as airway remodeling, persistent airway inflammation, and chronic allergy symptoms, in patients with perennial AR or asthma.^6^^,^^7^ Sensitization to fungi and bacteria is also associated with the progression of asthma severity.^8^, ^9^, ^10^, ^11^ Collectively, these findings emphasize the importance of accurately identifying causative allergens to facilitate the precise management of allergic diseases, including allergen immunotherapy, anti-IgE biologic therapy, environmental control measures, and elimination diets.^6^, ^7^, ^8^^,^^12^

The clinical guidelines and official statements of the European Academy of Allergy and Clinical Immunology recommend skin prick testing (SPT) and serological allergen-specific IgE assays as standard diagnostic approaches for identifying sensitization to environmental allergens.^6^^,^^7^^,^^12^ SPT is widely considered as advantageous as it is simple for trained personnel to use and can provide rapid results.^12^^,^^13^ SPT exhibits a relevant accuracy in terms of sensitization to aeroallergens. However, it has several limitations. These include the temporal discontinuation of medications to modify skin reaction and restricted applicability in patients with limited available skin surface due to the presence of active skin diseases, such as urticaria and atopic dermatitis.^13^

In vitro assays for detecting allergen-specific IgE antibodies are well-established alternatives to SPT.^12^ These assays quantify allergen-specific IgE in the serum using enzyme-linked immunoassay techniques based on allergen-IgE binding. ImmunoCAP (Thermo Fisher Scientific/Phadia, Uppsala, Sweden) and Immulite Immunoassay Systems (Siemens Healthcare Diagnostics, Erlangen, Germany) are widely used measuring units that provide a high reproducibility and facilitate the quantitative measurement of IgE levels.^12^^,^^14^, ^15^, ^16^, ^17^, ^18^ These systems have good concordance with SPT for several aeroallergens.^17^, ^18^, ^19^, ^20^ Nevertheless, they have several limitations, including allergen-dependent variability in sensitivity, the requirement for relatively large serum volumes, prolonged turnaround time, and the need for substantial laboratory infrastructures.^13^^,^^21^ Consequently, their use is generally limited to specialized hospitals and commercial diagnostic laboratories. In addition, in vitro IgE assays and SPT are not completely interchangeable because they rely on distinct biological principles and analytical methodologies.

Oriton IgE CHEMIPHAR™ (CHEMIPHAR; Nippon Chemiphar Co., Tokyo, Japan) is a commercially available serological allergen-specific IgE assay system in Japan that comprises the Oriton IgE kit and the NC-DP3000 analyzer. This system simultaneously measures up to 60 allergen-specific IgE antibodies using a sandwich enzyme immunoassay with a filter-based solid phase. The analytical detection range is 0.35–100 IU/mL, with values ≥ 0.35 IU/mL indicating positivity and ≥0.7 IU/mL suggesting clinical positivity. Owing to its rapid processing time (1 measurement in 12 min), compact instrumentation, and ease of operation, CHEMIPHAR may be suitable for routine clinical use. However, as only small-scale studies have reported sensitivity and specificity comparable with those of conventional in vitro IgE assay systems, evidence regarding its clinical performance remains limited.^22^

Therefore, the current study evaluated the analytical performance and clinical relevance of CHEMIPHAR in adult patients with allergic airway diseases by comparing its results with those of ImmunoCAP and SPT.

## Methods

### Participants

This retrospective observational study used data obtained from previous studies approved by the Institutional Review Board (approval numbers: 12–029, 14-008, and 2025-127). Patients aged ≥19 years who had asthma and/or AR were enrolled at the study center between June 2012 and March 2014 and between July 2014 and April 2021.

Asthma was diagnosed according to the Japanese guidelines for adult asthma,^7^ which includes a clinical history of asthma symptoms and either a ≥12% increase in baseline forced expiratory volume in 1 s (FEV_1_) after inhalation of 200 μg of salbutamol aerosol or the presence of bronchial hyper-responsiveness, defined as a methacholine PC20 < 4 mg/mL. AR was diagnosed by otolaryngologists in accordance with the Japanese guidelines for AR,^6^ particularly a clinical history of rhinitis symptoms persisting for >3 months.

The exclusion criteria included patients with asthma exacerbation or respiratory infection within 1 month prior to enrollment, those who received treatment with systemic corticosteroids or immunosuppressants, those who used medications that could influence SPT results, pregnant women, and those with severe comorbid conditions such as malignancy, severe renal failure, severe heart failure, and neurological disorders.

### Study design

In the original studies, the medical records of eligible patients were reviewed, and blood samples were collected. SPT was performed on the day of the outpatient visit. Blood samples were stored at −20 °C until analysis. SPT was performed on the forearms free of active skin disease, and medications that can affect SPT results were temporarily discontinued prior to testing. Patients with skin diseases were examined only if conditions such as atopic dermatitis and urticaria were clinically stable.

The serum allergen-specific IgE antibodies against 10 aeroallergens—Japanese cedar pollen (JCP), *Dermatophagoides farinae* (Df), ragweed, orchard grass, mugwort, *Alternaria*, *Aspergillus*, *Candida*, cat dander, and dog dander—were measured using CHEMIPHAR and ImmunoCAP. Fractional exhaled nitric oxide was measured in patients with asthma based on the American Thoracic Society recommendations.^23^

The Institutional Review Board approved the current study (approval numbers: 12–029, 14-008, and 2025-127). The need for a written informed consent was waived, and an opt-out option was provided on the hospital website.

### CHEMIPHAR assay

All procedures were automatically performed using the NC-DP3000 system with the Oriton IgE kit (Nippon Chemiphar Co., Tokyo, Japan), according to the manufacturer's instructions. In brief, allergen-specific IgE in the patient's serum reacted with biotinylated allergens, and the allergen-IgE complexes were captured by antibiotin antibodies immobilized on a filter. A secondary enzyme-labeled antibody was then bound to the allergen-IgE complex. After washing, the substrate was added, and the peroxidase activity on the filter was measured. IgE concentrations were calculated using a standard curve.

Specific IgE levels were classified from class 0 (<0.35 IU/mL) to class 6 (≥100 IU/mL). Results were automatically generated and recorded by the system. Allergen sensitization was defined as an allergen-specific IgE level of ≥0.35 IU/mL, a conventional cutoff widely used in allergy and asthma research.^24^^,^^25^ Although the ImmunoCAP system can report allergen-specific IgE values from 0.1 IU/mL, this lower value represents the analytical reporting range rather than the predefined clinical cutoff for sensitization used in the present study.

### SPT

All eligible patients according to the established guidelines underwent SPT.^26^ Patients receiving medications that could modify SPT results discontinued their treatment with antihistamines, leukotriene receptor antagonists, and β_2_-agonists at least 1 week prior to testing. SPT was conducted by trained allergy nurses or physicians and evaluated by a certified allergologist.

All pricks were performed on the volar aspect of the forearm. After skin disinfection with alcohol, 1 drop of each allergen extract and a negative control was placed on the skin. Then, the skin was gently pricked through each drop using a fine metal lancet. Wheal and flare responses were measured after 15 min. The same 10 allergen extracts (Torii Pharmaceutical Co., Tokyo, Japan) were used as those tested using CHEMIPHAR and ImmunoCAP. Physiological saline was utilized as the negative control. A wheal diameter ≥3 mm indicated a positive reaction.^26^

### ImmunoCAP

Allergen-specific IgE levels were measured using ImmunoCAP in a subset of eligible patients. Testing was performed by a commercial laboratory (BML Inc., Tokyo, Japan). IgE concentrations were classified from class 0 (<0.35 IU/mL) to class 6 (≥100 IU/mL). Allergen sensitization was defined using the same criteria as in the CHEMIPHAR assay.

### Statistical analysis

The primary objective was to evaluate whether CHEMIPHAR could be an alternative to SPT by demonstrating performance comparable to that of ImmunoCAP. The prevalence of allergen sensitization was compared among CHEMIPHAR, ImmunoCAP, and SPT. The concordance with SPT, sensitivity, specificity, positive predictive value (PPV), and negative predictive value (NPV) were calculated for CHEMIPHAR and ImmunoCAP. Allergen-specific IgE levels ≥0.35 IU/mL indicated positivity. As an additional sensitivity analysis, ImmunoCAP positivity was also evaluated using a cutoff of 0.1 IU/mL.

The agreement between assays and SPT was assessed using the Cohen's kappa (κ) statistic.^27^ Kappa values were interpreted as follows: almost perfect, 0.81–0.99; substantial, 0.61–0.80; moderate, 0.41–0.60; fair, 0.21–0.40; slight, 0.01–0.20; chance agreement, 0; and less than chance, <0.

The discriminative performance for SPT positivity was evaluated via receiver operating characteristic (ROC) curve analysis. The area under the curve (AUC) values were on an exploratory basis compared between CHEMIPHAR and ImmunoCAP in patients who completed all three tests. An AUC of 0.5 indicated no discriminative ability.

Analyses were conducted using all available data for each comparison, resulting in different sample sizes according to the specific comparison being performed. Normality was assessed using the Kolmogorov–Smirnov test. Parametric continuous variables were compared using the Student's *t*-test or analysis of variance. Nonparametric variables were compared using the Mann–Whitney *U* test or the Kruskal–Wallis test. Categorical variables were compared using the χ^2^ test or Fisher's exact test, as appropriate. Data were presented as mean ± standard deviation or median (25th–75th percentile), depending on distribution.

## Result

### Characteristics of the participants

CHEMIPHAR, ImmunoCAP, and SPT were performed in 168, 108, and 139 patients, respectively. Among these patients, 139 underwent CHEMIPHAR and SPT and 69 ImmunoCAP and SPT. Further, 79 completed all 3 examinations. More than 70% of patients had asthma, and approximately 80% had AR in each examination group. Approximately 76.4% of patients had asthma with comorbid AR. The CHEMIPHAR and ImmunoCAP groups did not significantly differ in terms of demographic characteristics (Table 1).

**Table 1:** Characteristics of the patients.

	CHEMIPHAR	Skin prick test	ImmunoCAP	p value
N	168	139	108	
Age, years	50 (35–64)	52 (36–64)	52 (39–64)	N. S.
Sex (%), (male/female, n)	46.4 (78/90)	46.8 (65/74)	49.1 (53/55)	N. S.
Smoking status, nonsmoking/past/current, n	99/54/15	82/47/10	59/38/11	N. S.
BMI (m^2^/kg)	23.0 (21.0–26.0)	23.0 (20.0–26.0)	23.0 (20.0–26.0)	N. S.
Asthma, n (%)	121 (72.0)	99 (71.2)	82 (75.9)	N. S.
Allergic rhinitis, n (%)	134 (79.8)	110 (79.1)	86 (79.6)	N. S.
Blood eosinophil ratio, %	4.0 (2.4–6.4)	4.0 (2.5–6.5)	3.9 (2.2–7.5)	N. S.
Blood eosinophil count, cells/μL	258 (138–428)	270 (136–436)	269 (141–454)	N. S.
Total blood IgE level, IU/mL	195.0 (65.0–531.5)	186.0 (57.5–186.0)	197.0 (65.0–512.0)	N. S.
FeNO^a^, ppb	26 (13–16)	28 (12–54)	26 (13–47)	N. S.

CHEMIPHAR: Oriton IgE CHEMIPHAR^TM^, FeNO: fraction of exhaled nitric oxide, N.S.: nonsignificant.

The nonparametric data were expressed as median (25%–75%), unless otherwise indicated.

a Values were measured only in patients with asthma

### Prevalence of sensitization to inhalant allergens

All 3 methods had the highest prevalence of sensitization to JCP (CHEMIPHAR, 74.4%; ImmunoCAP, 73.5%; and SPT, 63.2%), followed by *Df* (51.8%, 47.5%, and 56.4%, respectively) (Fig. 1). Sensitization to ragweed (27.7%, 28.4%, and 16.5%) and orchard grass (27.5%, 39.1%, and 23.3%) exhibited a moderate prevalence across methods. CHEMIPHAR and ImmunoCAP had a significantly higher prevalence of sensitization to ragweed, compared with SPT. ImmunoCAP had a significantly higher prevalence of sensitization to orchard grass than CHEMIPHAR and SPT.

**Fig. 1:**
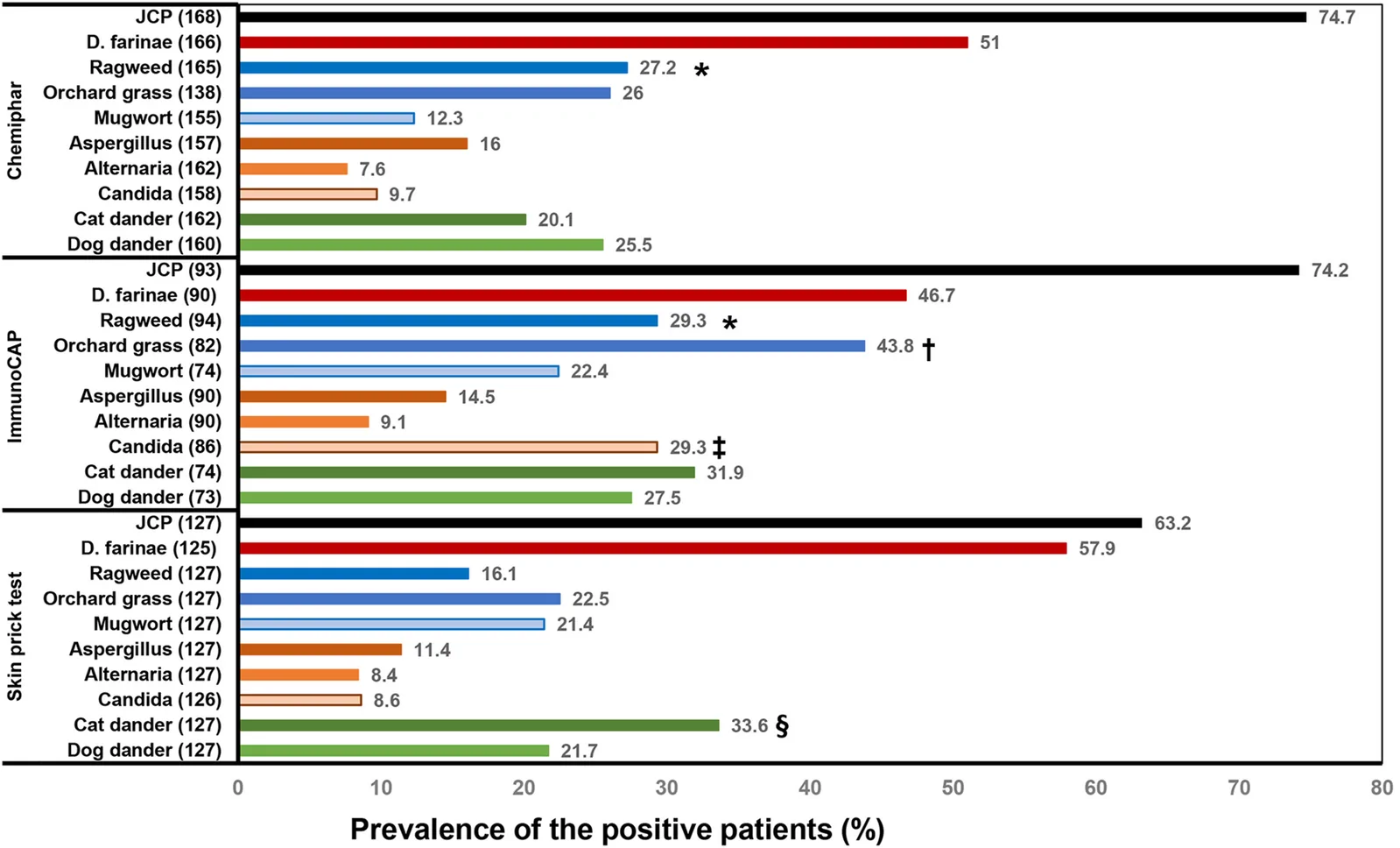
Proportion of patients with positive CHEMIPHAR, ImmunoCAP, and SPT results. Variables were analyzed using the *X*^2^ test. ⁎; p = 0.041, vs SPT, ^†^; p = 0.033, vs CHEMIPHAR and SPT, ^‡^; p = 0.012, vs CHEMIPHAR, ^§^; p = 0.039, vs SPT. SPT: skin prick test, JCP: Japanese cedar pollen, *D. farinae*: *Dermatophagoides farinae.*

ImmunoCAP presented with a significantly higher prevalence of sensitization to mugwort than CHEMIPHAR (26.8% and 11.6%, respectively). Sensitization to *Candida* was modestly but significantly more prevalent with ImmunoCAP (16.3%) than with SPT (6.0%). All 3 methods had a low prevalence of sensitization to *Aspergillus* (CHEMIPHAR, 15.9%; ImmunoCAP, 13.0%; and SPT, 10.5%, respectively) and *Alternaria* (7.4%, 13.0%, and 8.3%, respectively). Moderate prevalence was observed for cat dander (25.0%, 26.2%, and 31.6%, respectively) and dog dander (25.5%, 23.2%, and 20.3%, respectively).

### Diagnostic performance of CHEMIPHAR and ImmunoCAP for detecting SPT results

CHEMIPHAR had rates of concordance to SPT exceeding 80% for most inhalant allergens, except for cat dander (approximately 70%) (Table 2). The sensitivity varied by allergen. In particular, it was the highest for JCP (approximately 100%), followed by *Df* and orchard grass (>70%). Further, it was moderate for ragweed, *Candida* and dog dander (50%–65%), and lower for the remaining allergens (30%–44%). The specificity exceeded 85% for all allergens except JCP (>70%). The PPVs were high for JCP, *Df*, orchard grass, and mugwort (84.6%–91.2%), moderate for *Alternaria* and pet dander (50%–71%), and low for the remaining allergens (30%–46%). The NPVs were approximately 90% for most allergens, except for *Df* and pet dander (>70%).

**Table 2:** Performance of CHEMIPHAR and ImmunoCAP in agreement with the skin prick test.

	CHEMIPHAR						ImmunoCAP					
	Number	Sensitivity	Specificity	PPV	NPV	Concordance	Number	Sensitivity	Specificity	PPV	NPV	Concordance
JCP	124	97.4	71.7	85.4	94.3	87.9	67	89.5	62.1	75.6	81.8	77.6
*Df*	120	75.4	90.2	91.2	73.0	81.7	65	70.3	100.0	100.0	71.8	83.1
Ragweed	122	65.0	85.3	46.4	92.6	82.0	66	72.7	85.5	50.0	94.0	82.1
Orchard grass	102	73.9	96.2	85.0	92.7	91.2	57	73.9	96.2	85.0	92.7	91.2
Mugwort	116	44.0	97.2	84.6	93.8	86.2	52	71.4	86.7	45.5	95.1	84.6
Alternaria	118	30.0	97.3	50.0	91.5	91.8	65	50.0	98.3	75.0	95.1	93.8
Aspergillus	113	38.5	90.0	33.3	91.8	84.1	64	71.4	94.7	62.5	96.4	92.2
Candida	119	50.0	93.6	30.0	91.3	91.6	63	100.0	88.3	30.0	100.0	90.5
Cat dander	120	36.6	92.4	71.4	73.7	73.3	50	33.3	91.4	62.5	76.2	74.0
Dog dander	119	61.5	90.3	64.0	89.4	84.0	47	50.0	92.3	57.1	90.0	87.2

CHEMIPHAR: Oriton IgE CHEMIPHAR ^TM^, Df: Dermatophagoides farinae, JCP: Japanese cedar pollen, NPV: negative predictive value, PPV: positive predictive value

ImmunoCAP also showed rates of concordance to SPT exceeding 80% for most allergens, except for JCP and cat dander (approximately 70%) (Table 2). The sensitivity ranged from moderate to extremely high, with the highest sensitivity observed for *Candida* (100%), followed by JCP, *Df*, ragweed, orchard grass, mugwort and *Aspergillus* (>70%). The specificity exceeded 85% for all allergens except JCP (approximately 60%). The PPVs were generally moderate (45.5%–62.5%), with higher values for JCP, *Df*, orchard grass, and Alternaria (>75%), and lower values for *Candida* (approximately 30%). The NPVs were high for all allergens (70%–100%). Quantitative results of CHEMIPHAR, ImmunoCAP, and SPT for each allergen are summarized in Supplemental Table 1.

### ROC analysis

The ROC analysis demonstrated a significant discriminative performance for both CHEMIPHAR and ImmunoCAP in identifying SPT positivity for most inhalant allergens. CHEMIPHAR had AUC values > 0.90 for *Df* and orchard grass, >0.80 for JCP, ragweed, mugwort, and *Candida* and >0.70 for *Aspergillus* and cat and dog dander. ImmunoCAP exhibited AUC values > 0.90 for orchard grass and *Candida*, >0.80 for JCP and *Df*, and >0.70 for ragweed and *Aspergillus* (Fig. 2). Meanwhile, the AUC values for the remaining allergens were not significant.

**Fig. 2:**
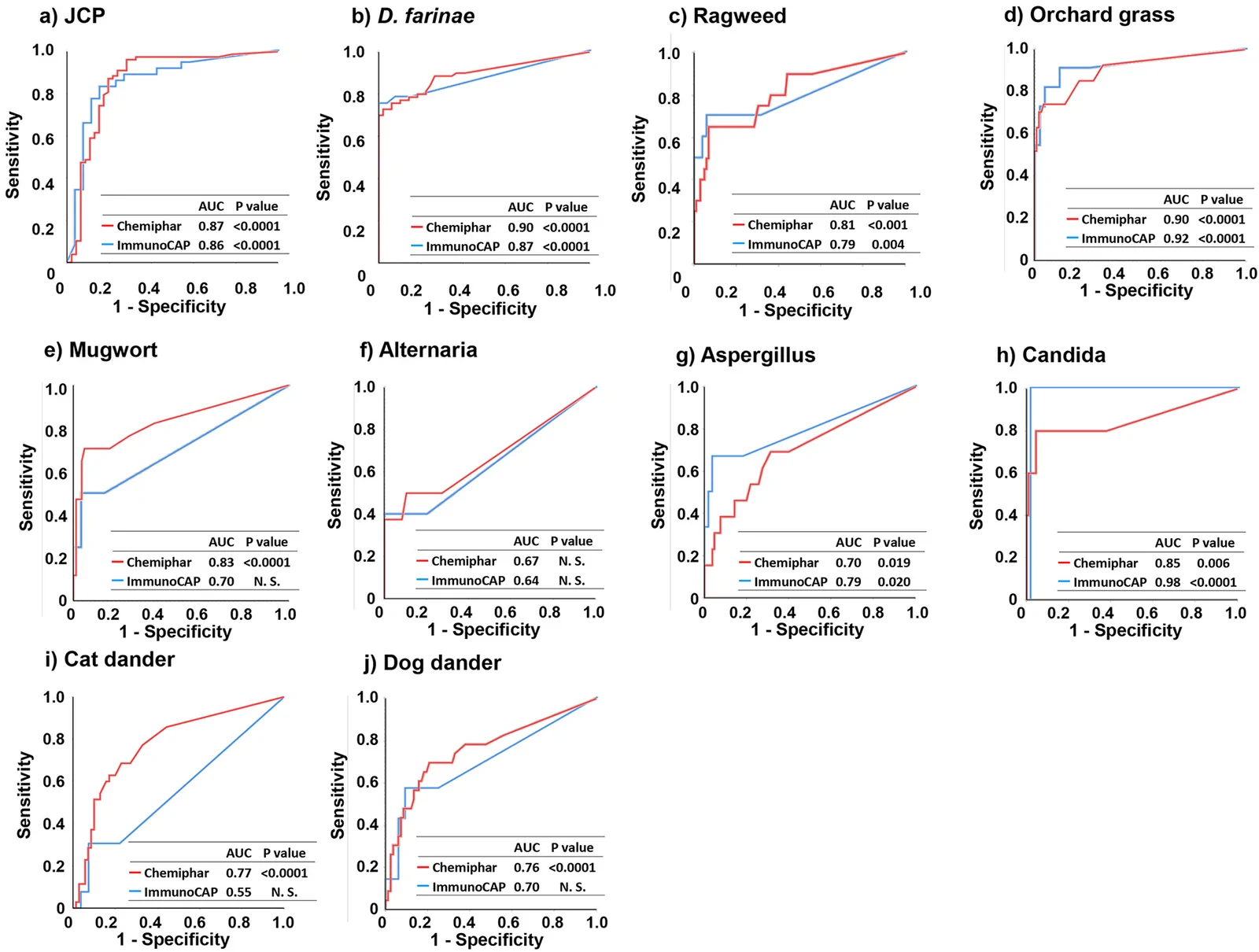
Receiver operating characteristic curve analysis for CHEMIPHAR and ImmunoCAP to identify SPT positivity. The AUCs were similar between CHEMIPHAR and ImmunoCAP. AUC, area under the curve, *D. farinae*: *Dermatophagoides farinae*.

In patients who completed all 3 examinations, the AUC values were generally consistent with those observed in the overall cohort, except for fungi in CHEMIPHAR and *Candida* in ImmunoCAP (Supplementary Fig. 1). CHEMIPHAR had a significantly higher AUC for cat dander than ImmunoCAP (p = 0.03, n = 32), whereas the AUC values for the other allergens were comparable between the 2 systems.

### Agreement analysis

CHEMIPHAR demonstrated significant Kappa coefficients, which were as follows: substantial agreement with SPT for JCP, *Df*, and orchard grass; moderate agreement for ragweed, mugwort, and dog dander; and fair agreement for *Alternaria*, *Aspergillus*, *Candida*, and cat dander (Table 3).

**Table 3:** Inter-agreement analysis of CHEMIPHAR and ImmunoCAP with SPT.

	CHEMIPHAR				ImmunoCAP			
	Number	Kappa coefficient	95% CI	p value	Number	Kappa coefficient	95% CI	p value
JCP	124	0.70	0.59–0.85	< 0.0001	67	0.52	0.32–0.74	< 0.0001
*Df*	120	0.64	0.50–0.77	< 0.0001	65	0.67	0.56–0.79	< 0.0001
Ragweed	122	0.43	0.24–0.63	0.001	66	0.49	0.24–0.75	0.001
Orchard grass	102	0.74	0.57–0.90	< 0.0001	57	0.74	0.39–0.89	< 0.0001
Mugwort	116	0.51	0.30–0.71	< 0.0001	52	0.47	0.15–0.78	< 0.0001
Alternaria	118	0.33	0.02–0.65	< 0.0001	65	0.57	0.18–0.95	< 0.0001
Aspergillus	113	0.27	0.02–0.52	0.004	64	0.62	0.32–0.93	0.004
Candida	119	0.33	0.01–0.65	< 0.0001	63	0.42	0.08–0.75	< 0.0001
Cat dander	120	0.33	0.19–0.46	< 0.0001	50	0.28	−0.01–0.57	0.032
Dog dander	119	0.53	0.34–0.72	< 0.0001	47	0.50	0.14–0.85	0.001

CHEMIPHAR: Oriton IgE CHEMIPHAR ^TM^, CI: confidence interval, Df: Dermatophagoides farinae, JCP: Japanese cedar pollen

Similarly, ImmunoCAP had significant Kappa coefficients, which were as follows: substantial agreement for *Df*, orchard grass, and *Aspergillus*; moderate agreement for JCP, ragweed, mugwort, *Alternaria*, *Candida*, and dog dander; and fair agreement for cat dander.

### Sensitivity analysis using an ImmunoCAP cutoff of 0.1 IU/mL

In an additional sensitivity analysis using an ImmunoCAP cutoff of 0.1 IU/mL, the prevalence of sensitization increased modestly for several allergens. Sensitivity was generally similar or slightly increased, whereas specificity, PPV, NPV, and overall concordance with SPT decreased for several allergens. The kappa coefficients were slightly higher for JCP and Df but lower for most other allergens. Overall, the 0.1 IU/mL cutoff did not significantly improve agreement with SPT compared with the predefined cutoff of 0.35 IU/mL (Supplementary Fig. 2 and Supplementary Tables 2 and 3).

## Discussion

In this study, CHEMIPHAR had a prevalence of sensitization to major environmental allergens comparable to that determined by SPT and performance equivalent to ImmunoCAP in predicting SPT results in patients with allergic airway diseases. CHEMIPHAR also exhibited an acceptable agreement with SPT for *Df*, JCP, several pollens, and pet dander. SPT reflects IgE-mediated allergic reactions promoted by mast cells in response to causative allergens. Therefore, these findings indicate that CHEMIPHAR is a clinically feasible in vitro method for identifying sensitization to environmental allergens. In particular, CHEMIPHAR offers potential advantages over SPT in terms of independence from skin conditions and concomitant medications. These characteristics may facilitate its use in routine clinical practice.

CHEMIPHAR had sensitization frequencies for JCP and *Df* similar to those obtained with ImmunoCAP, which are the most prevalent causative allergens in allergic airway diseases in Japan. These findings are consistent with those of nationwide surveys of serum allergen-specific IgE in Japan, which reported the highest sensitization rates for JCP, followed by house dust mites, and relatively low prevalence rates of fungal sensitization.^28^^,^^29^ Taken together, these data support the ability of CHEMIPHAR to provide epidemiologically plausible sensitization profiles. In contrast, ImmunoCAP presented with higher sensitivity for several allergens, including ragweed, orchard grass, mugwort, *Candida*, and Aspergillus, which may reduce the risk of false-negative results. Therefore, ImmunoCAP may be preferred when evaluating sensitization to selected allergens.

Both CHEMIPHAR and ImmunoCAP demonstrated good discriminatory ability for predicting SPT positivity, as reflected by high AUC values for several inhalant allergens, which is in accordance with previous reports.^20^^,^^30^^,^^31^ However, differences in AUC values were observed between the 2 systems for certain allergens. In the subgroup of patients who completed all 3 examinations, CHEMIPHAR demonstrated significant discriminative ability for several inhalant allergens. However, the overall performance of the 2 assays was generally comparable. Considering its shorter turnaround time, CHEMIPHAR is likely a convenient method for screening allergen sensitization.

In contrast to the plausible predictivity and substantial agreement of CHEMIPHAR and ImmunoCAP for major allergens, CHEMIPHAR showed a fair agreement with SPT for *Aspergillus*, *Alternaria*, *Candida*, and pet dander. Meanwhile, ImmunoCAP had moderate to substantial agreement for some of these allergens. Such discrepancies may reflect differences in assay performance, allergen extract composition, and cross-reactivity among crude fungal antigens. Previous studies have reported considerable cross-reactivity among fungal extracts, which may not always represent true clinical sensitization.^32^, ^33^, ^34^ Therefore, the assessment of fungal sensitization should incorporate clinical history, exposure patterns, and, if necessary, multiple diagnostic modalities. Laboratory-based IgE testing alone may be insufficient for the accurate identification of fungal sensitization.

This study has several limitations. First, the number of patients undergoing ImmunoCAP testing was relatively small, which might have limited statistical power for some comparisons. Nevertheless, the consistency of our ImmunoCAP findings with that of previous studies supports the robustness of the current results. Second, allergen components were not analyzed. Therefore, the potential influence of panallergen-mediated cross-reactivity could not be assessed. Further studies incorporating component-resolved diagnostics may provide additional insights into the causes of discordant results among different allergy testing methods. Finally, the present study was not designed to compare the economic aspects of CHEMIPHAR and ImmunoCAP. Therefore, no conclusions regarding the relative cost-effectiveness of the 2 assays can be drawn from the present data. Future studies evaluating operational and economic considerations may further clarify the practical advantages of each assay.

In conclusion, CHEMIPHAR had a clinically relevant performance in identifying sensitization to inhalant allergens, and it showed good agreement with SPT in patients with allergic airway diseases. Considering that the current guidelines recognize SPT as the reference standard and ImmunoCAP as a reliable alternative, the comparable performance of CHEMIPHAR supports its potential utility as a diagnostic tool for assessing allergen sensitization and predicting SPT outcomes in clinical practice.

## Abbreviations

AR, allergic rhinitis; AUC, area under the curve; CHEMIPHAR, Oriton IgE CHEMIPHAR™; *Df, Dermatophagoides farinae;* FEV1, forced expiratory volume in 1 s; IgE, immunoglobulin E; JCP, Japanese cedar pollen; NPV, negative predictive value; PC20, provocative concentration causing a 20% decrease in FEV1; PPV, positive predictive value; ROC, receiver operating characteristic; SPT, skin prick test.

## Ethics statement

This study was approved by the Institutional Review Board of Saitama Medical University Hospital. The need for a written informed consent was waived, and an opt-out option was provided on the hospital website.

## Author contribution

EN performed the experiments and statistical analysis, and drafted the manuscript. TS conducted and organised the study, performed the statistical analysis, and drafted the manuscript. RS and YU contributed to data collection. KN and TK contributed to the interpretation of the results. MN advised on and organised the study and interpreted the results. All authors discussed the results and implications and have read and approved the final manuscript.

## Consent of publication

All authors read and approved the final manuscript to submit World Allergy Organization Journal.

## Availability of data and materials

Data and scripts for analysis that were used in this research will be available upon request to the corresponding author.

## Disclosure of the use of generative AI and AI-assisted technologies

During the preparation of this manuscript, the authors used ChatGPT (OpenAI) to assist with language editing and improving the clarity and readability of the text. The authors reviewed and edited the output as necessary and take full responsibility for the content of the publication.

## Funding

No funding has been received for this manuscript.

## Conflicts of interest

All authors declare the following: payment/service information. All authors declare that no financial support was received from any organization for the submitted work. Financial relationships: Tomoyuki Soma, Kazuyuki Nakagome, Yoshitaka Uchida, and Makoto Nagata declare receiving personal fees from AstraZeneca, GSK, Sanofi, and Novartis Pharma. Other relationships: All authors declare that there are no other relationships or activities that could appear to have influenced the submitted work.

## Figures and Tables

**figs1:**
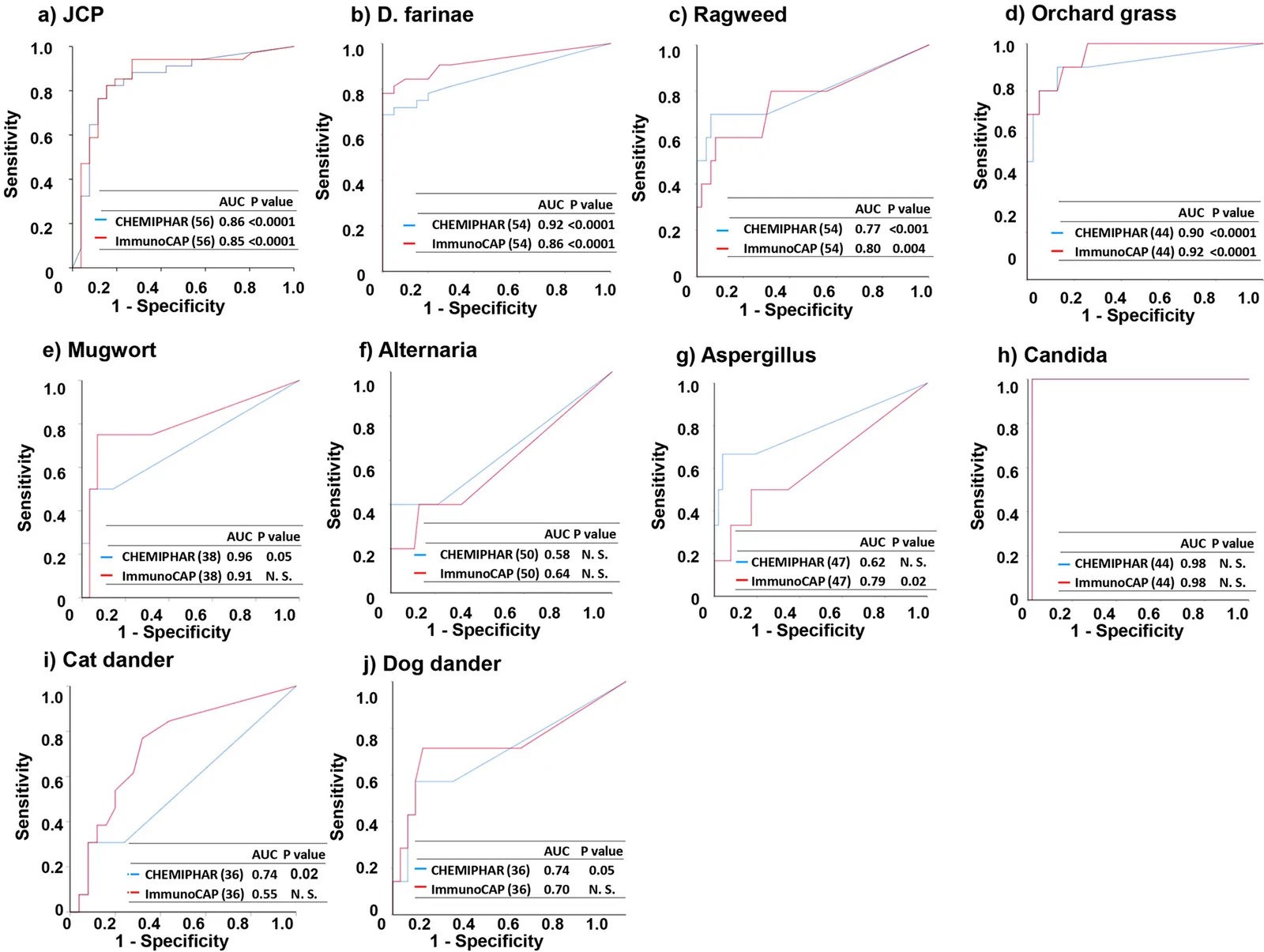


**figs2:**