# Supplementary material for: Clinical utility of Oriton IgE CHEMIPHAR™ for predicting skin prick test positivity in patients with allergic airway diseases
Source: World Allergy Organ J. 2026 Nov 2;19(11):101479. doi: 10.1016/j.waojou.2026.101479 (PMC13755474; doi:10.1016/j.waojou.2026.101479)
Supplement: Supplementary file 1 [file mmc1.docx]

Supplementary Table 1. Magnitude of allergen-specific IgE responses and skin prick test reactivity

|  | CHEMIPHAR (IU/mL) | ImmunoCAP (IU/mL) | Skin prick test (mm) |
| --- | --- | --- | --- |
| JCP | 18.88 (13.90–23.87) | 22.31 (16.30–28.33) | 5.5 (4.6–6.5) |
| *Df* | 17.65 (12.11–23.19) | 13.53 (7.98–19.08) | 6.7 (5.4–7.9) |
| Ragweed | 1.40 (0.56–2.24) | 1.34 (0.67–2.01) | 1.7 (1.1–2.3) |
| Orchard grass | 4.26 (1.97–6.52) | 5.53 (2.42–8.64) | 2.4 (1.7–3.2) |
| Mugwort | 0.37 (0.10–0.66) | 0.59 (0.17–1.01) | 2.3 (1.6–3.1) |
| Alternaria | 0.36 (0.01–0.71) | 0.42 (0.04–0.80) | 1.2 (0.6–1.7) |
| Aspergillus | 0.66 (0.20–1.12) | 0.80 (0.00–1.66) | 1.3 (0.7–1.8) |
| Candida | 0.70 (0.17–1.23) | 1.61 (0.40–2.82) | 1.5 (0.8–2.1) |
| Cat dander | 1.82 (0.89–2.75) | 2.16 (0.57–3.75) | 2.7 (2.0–3.4) |
| Dog dander | 2.91 (0.96–4.86) | 3.62 (0.73–6.51) | 2.0 (1.4–2.6) |

Data are presented as mean values with 95% confidence intervals to illustrate the magnitude of allergen-specific IgE responses and SPT reactivity for each allergen.

CHEMIPHAR: Oriton IgE CHEMIPHAR^TM^, *Df*: *Dermatophagoides farinae*, JCP: Japanese cedar pollen

Supplementary Table 2. Performance of ImmunoCAP in agreement with the skin prick test using the cutoff of 0.10 IU/mL

|  | ImmunoCAP (0.10 IU/mL) | | | | |
| --- | --- | --- | --- | --- | --- |
|  | Sensitivity | Specificity | PPV | NPV | Concordance |
| JCP (N = 67) | 94.7 | 55.2 | 73.5 | 88.9 | 77.6 |
| *Df* (N = 65) | 78.4 | 92.9 | 95.5 | 76.5 | 84.6 |
| Ragweed (N = 66) | 72.4 | 76.4 | 38.1 | 93.3 | 75.8 |
| Orchard grass (N = 57) | 90.0 | 85.3 | 64.3 | 96.7 | 85.9 |
| Mugwort (N = 52) | 71.4 | 86.7 | 45.5 | 95.1 | 86.8 |
| Alternaria (N = 65) | 50.0 | 91.5 | 37.5 | 94.7 | 87.7 |
| Aspergillus (N = 64) | 71.4 | 93.0 | 55.6 | 96.4 | 90.6 |
| Candida (N = 63) | 100.0 | 83.3 | 23.1 | 100.0 | 84.1 |
| Cat dander (N = 50) | 33.3 | 88.6 | 55.6 | 75.6 | 72.0 |
| Dog dander (N = 47) | 62.5 | 82.1 | 41.7 | 91.4 | 78.7 |

CHEMIPHAR: Oriton IgE CHEMIPHAR ^TM^, *Df*: *Dermatophagoides farinae*, JCP: Japanese cedar pollen, NPV: negative predictive value, PPV: positive predictive value

Supplementary Table 3. Inter-agreement analysis of ImmunoCAP with SPT using the cutoff of 0.10 IU/mL

|  | ImmunoCAP (0.10 IU/mL) | | |
| --- | --- | --- | --- |
|  | Kappa coefficient | 95% CI | p value |
| JCP (N = 67) | 0.67 | 0.32–0.73 | <0.0001 |
| *Df* (N = 65) | 0.69 | 0.53–0.86 | <0.0001 |
| Ragweed (N = 66) | 0.36 | 0.12–0.61 | <.0001 |
| Orchard grass (N = 57) | 0.56 | 0.32–0.79 | <0.0001 |
| Mugwort (N = 52) | 0.43 | 0.12–0.74 | 0.001 |
| Alternaria (N = 65) | 0.36 | 0.00–0.72 | <0.0001 |
| Aspergillus (N = 64) | 0.57 | 0.26–0.89 | <0.0001 |
| Candida (N = 63) | 0.32 | 0.03–0.61 | 0.001 |
| Cat dander (N = 50) | 0.24 | -0.05–0.54 | N. S. |
| Dog dander (N = 47) | 0.41 | 0.10–0.73 | 0.003 |

CHEMIPHAR: Oriton IgE CHEMIPHAR ^TM^, CI: confidence interval, *Df*: *Dermatophagoides farinae*, JCP: Japanese cedar pollen
